# Intention, magnitude and factors associated with bottle feeding among mothers of 0–23 months old children in Holeta town, Central Ethiopia: a cross sectional study

**DOI:** 10.1186/s40795-017-0174-y

**Published:** 2017-07-05

**Authors:** Tadesse Kebebe, Hirut Assaye

**Affiliations:** 1 Ethiopian Public Health Institute, Addis Ababa, Ethiopia; 2 Department of Applied Human Nutrition, Bahir Dar University, Bahir Dar, Ethiopia

**Keywords:** Bottle feeding, Intention, Holeta, Ethiopia

## Abstract

**Background:**

World Health Organization (WHO) recommends that bottle feeding should be avoided for infant and young child feeding since it has an impact on optimal breastfeeding, appropriate complementary feeding and bottles with a nipple are prone to contamination. The objectives of this study were to determine intention, magnitude and factors associated with bottle feeding among mothers of 0–23 months infants and children.

**Methods:**

Community based cross sectional study was conducted from February to May 2016. A total of 422 mothers who had children 0–23 months were included in the study. Systematic random sampling was used to select the study subjects. Data were collected using a pre-tested interviewer administered structured questionnaire. The data were cleaned, coded, entered in to EPI-INFO version 3.5.4, and transferred and analyzed using SPSS. Odds ratio was calculated with 95% CI to identify factors associated with bottle feeding practice. *P*-values less than 0.05 were considered as statistically significant.

**Results:**

The prevalence of bottle-feeding in this study was 19.6% and another 27.6% mothers have intention of bottle feeding. Being infant age of 0–5 months [AOR = 0.27;95% CI:(0.12,0.62)] and being a housewife [AOR = 0.37;95% CI:(0.21,0.67)] were negatively associated while having three under five children [AOR = 2.77;95% CI:(1.07,7.14)], not attending PNC follow-up [AOR = 2.13;95% CI:(1.19,4.97)], lower age of mothers [AOR = 3.38;95% CI:(1.48,7.73)] and not counseled on bottle feeding [AOR = 2.18;95% CI:(1.24,3.83)] were positively associated with bottle feeding.

**Conclusion:**

The prevalence of bottle feeding in the study area was high compared to the national prevalence of bottle feeding. Working outside home, lower maternal age, older age of children, having more than one under five children in the household, not attending PNC follow-up and not counseled on bottle feeding were found to be risk factors associated with bottle feeding practice in the study area.

## Background

Infant and young child feeding (IYCF) practices affect the nutritional status of children under 2 years of age and eventually impact child survival [1]. The feeding of newborn infants has important implications for immediate and future health especially in developing countries such as Ethiopia which have high rates of malnutrition, infectious diseases and mortality among children under the age of 5 years [2, 3].

The first 2 years of a child’s life are particularly important, as optimal nutrition during this period lowers morbidity and mortality, reduces the risk of chronic diseases, and fosters better development overall [4]. Around 32% of children less than 5 years of age in developing countries are stunted and 10% are wasted and the major reason for such high burden of malnutrition is sub optimal IYCF practice [5].

Optimal infant and young child feeding practices as recommended by the World Health Organization includes early initiation of breast feeding within an hour of birth; exclusive breastfeeding till 6 months of age; introduction of complementary feeding at 6 months while continuing breast-feeding up to 2 years or beyond and ensuring proper use of breast-milk substitutes. However, breast milk substitutes are used commonly worldwide with bottle feeding which should be avoided due to its impact on optimal breastfeeding and appropriate complementary feeding. Moreover, feeding bottles are associated with diarrheal disease morbidity and mortality as it is difficult to keep it clean especially in developing countries where sanitation is poor [1].

Avoidance of artificial teats or pacifiers is a crucial strategy to promote universal breastfeeding [6, 7]. Exposure of infants to artificial nipples (bottle feeding) has been strongly associated with breastfeeding problems [1, 6]. This is due to nipple confusion occurs when infants are exposed to two different feeding methods, bottle and breast, resulting in the infant refusing to breastfeed [6].

Infant and young child feeding practices in Ethiopia are suboptimal [8]. According to the 2011 Ethiopian Demographic and Health Survey (EDHS), only 52% of infants less than 6 months were exclusively breastfed. The 2011 EDHS also indicated that 13% of 0–23 months infants and young children and 16% of infants under-6 months were fed using a bottle with a nipple [8]. Even higher prevalence of bottle feeding (38%) was reported in some areas of the country such as Oromia region [9]. There seems also an increasing trend (11 to 13%) in bottle feeding practice in the country as reported in the two consecutive demographic and health surveys (EDHS, 2005; EDHS, 2011) [8, 10]. In general, a large shift has been observed from breastfeeding to bottle feeding in the urban areas of developing countries [8–10].

Although there are some reports or studies in Ethiopia on prevalence of bottle feeding and associated factors [11–15], there is no any study regarding bottle feeding from the study communities. Besides, research based information is lacking on intention despite the fact that intention is an important predictor to practice bottle feeding in the future.

Therefore, this study was carried out to determine intention, magnitude and factors associated with bottle feeding among mothers of 0–23 months infants and children who live in Holeta town, Central Ethiopia.

## Methods

### Study design, setting and study population

A community based cross-sectional study was conducted in Holeta town from February to March 2016. Holeta is a suburban area in Oromia region, and found 25 km away from Addis Ababa, the capital city of Ethiopia. The town has eight kebeles (smallest administrative unit in Ethiopia). Mothers or care givers of infants and young children (0–23 months) were included in the study.

### Sample size and sampling procedures

Sample size was determined using single population proportion formula with the following assumptions; Proportion, 50%; margin of error, 5; and 95% confidence interval. A sample size of 422 was taken after considering 10% non response rate. The total number of mothers or care givers of infants and young children (0–23 months) in each kebele was obtained from the town’s health posts. Then, sample size was proportionally allocated to each kebele and systematic random sampling was used to select study participants. The total number of mothers/caregivers in each kebele was divided by the allocated sample size to get the sampling interval.

### Variables of the study and key measures

The dependent variables were bottle feeding practice and intention to bottle feeding while independent variables included socio-demographic and economic characteristics and mother’s health service utilization such as history of ANC, place of delivery, PNC, counseling/advice on IYCF and information on bottle feeding is not recommended.

The prevalence of bottle feeding was assessed by World Health Organization definition of this indicator “proportion of children 0–23 months of age that were fed any liquid (including breast milk) or semisolid from bottle with nipple/teat in previous 24 hours prior to data collection period”. Intention to bottle feed was assessed by asking mothers “Do you have a plan to practice bottle feeding in the future?”.

### Data collection procedure

Data were collected using pre-tested and interviewer-administered questionnaire. Questionnaires were initially prepared in English language and then translated into local languages of community Afaan Oromo and Amharic and then translated back to English to check consistency of meaning.

For data collection, four urban health extension workers were hired as data collectors. Data collectors and supervisors were trained on the study objectives, purpose and interviewing techniques based on the research instrument. Pre- test was done on 10% of similar subjects. After the pretest, sections or questions which were cited as inconsistent, invalid and weekly constructed or were likely to give unreliable data were reviewed and corrected.

### Data analysis

The collected data were cleaned, coded, entered into EPI-INFO version 3.5.4 software and transported to SPSS version 20. The association between single explanatory variable and dependent variable was examined through bivariable analysis, by computing odds ratio at 95% confidence level.

Variables with *p*-value less than 0.2 were included in the multiple logistic regression analysis to identify factors associated with bottle feeding. For all statistical significance tests between each independent and dependent variables, significance level was fixed at *P*-value <0.05.

## Results

### Socio-demographic characteristics

A total of 418 respondents participated in this study with a response rate of 99.1%. The mean age of the mothers was 29.3 years with ±6.5 SD and the mean age of infants/children was 10.6 months with ±6.1 SD. About 51.9% of infants/children were females. Higher proportions of mothers (54.8%) were Orthodox Christians in religion and Oromo (64.8%) in ethnicity. About 46.4% of mothers had attended formal education. Regarding their occupation, 233 (55.7%) of mothers were housewives and majority of husbands had attended formal education 363 (94.9%) (Table 1).

**Table 1:** Socio-economic and demographic characteristics of respondents, Holeta Town, Central Ethiopia, 2016

Characteristics		Frequency	Percent (%)
Residence	Urban	275	65.8
	Rural	143	34.2
Role of respondents	Mother	373	89.2
	Care givers	45	10.8
Age of mother (in years)	15–24	115	27.5
	25–34	202	48.3
	> = 35	101	24.2
Sex of infant/child	Female	217	51.9
	Male	201	48.1
Age of child (in month)	0–5	111	26.5
	6–11	104	24.9
	12–23	203	48.6
Religion	Orthodox	229	54.8
	Protestant	131	31.3
	Muslim	58	13.9
Ethnicity	Oromo	271	64.8
	Amhara	83	19.9
	Gurage	45	10.7
	Others	19	4.6
Marital status	Married	384	91.9
	Single	8	1.9
	Others	26	6.2
Educational status of mother	No formal education	121	28.9
	Primary	194	46.4
	Secondary	77	18.4
	Above secondary	26	6.2
Occupational status of mother	Housewife	233	55.7
	Merchant/private Job	104	24.9
	Employed	54	12.9
	Others	27	6.5
Educational status of husband	No formal education	22	5.1
	Primary	170	40.7
	Secondary	140	33.5
	Above secondary	53	12.7
Number of under 5	One	91	21.8
	Two	263	62.9
	Three	64	15.3
House hold income (Ethiopian Birr)	<500	60	14.4
	500–999	115	27.5
	1000–1499	57	13.6
	> = 1500	186	44.5

### Health care related characteristics

Among the study participants, 390 (93.3%) and 343 (82.1%) had history of antenatal and postnatal follow up respectively. More than half of the respondents (62.7%) gave birth at health institutions and higher proportion (96.2%) of the respondents had received advice on infant and young child feeding. Among mothers who had received advice/counseling on infant and young children feeding, 230 (55.0%) stated that they were informed that bottle feeding is not recommended in IYCF practice during the counseling session (Table 2).

**Table 2:** Health care related characteristics of study participants, Holeta Town, central Ethiopia, 2016

Characteristics		Frequency	Percent
Antenatal Care(ANC)	Yes	390	93.3
	No	28	6.7
Place of birth	Health Institutions	262	62.7
	Home	156	37.3
Postnatal care(PNC)	Yes	343	82.1
	No	75	17.9
Had received advice/counseling on IYCF	Yes	402	96.2
	No	16	3.8
Informed about bottle feeding is not recommended in IYCF during counseling	Yes	230	55.0
	No	188	45.0

### Bottle feeding practice

The prevalence of bottle-feeding practice in the study area was 19.6% and the prevalence was 8.1, 22.1 and 24.6% when disaggregated into age categories; 0–5 month, 6–11 month and12–23 months respectively. Among respondents who bottle fed their infants/children, 71 (86.6%) used cow milk and 24 (29.3%) were infants under 1 year. Majority of bottle feeding mothers/caregivers 72 (87.6%) indicated the convenience/easiness of bottle feeding as a major reason to practice it. Other reasons mentioned for practicing bottle feeding by study participants were the following; good to promote growth (19.5%), busy for work outside home (58.5%), and insufficient breast milk (26.8%) (Table 3).

**Table 3:** Bottle feeding practice among study participants, Holeta Town, Central Ethiopia, 2016

Characteristics		Frequency	%
Prevalence of bottle feeding (0-23 months)	Yes	82	19.6
	No	336	80.4
Foods used for bottle feeding	Cow milk	71	86.6
	Formula	11	13.4
	Cow milk for <1 year infants	24	29.3
	Bottle feed under 6-months infants	9	11
Reasons for practicing bottle feeding	Convenient/easy	72	87.8
	Good to promote growth	16	19.5
	Busy for work outside home	48	58.5
	Insufficient breast milk	22	26.8

### Intention to practice bottle feeding

Participants who were not practicing bottle feeding during the study period were asked if they have an intention to practice bottle feed their infants or children in the future. In this regard, 93(27.6%) of the respondents had intention to bottle feed and the intention to bottle feed by majority of the mothers (81.70%) was to get help from other care givers or family members for feeding the child (Table 4).

**Table 4:** Intention to practice bottle feeding among study participants, Holeta Town, Central Ethiopia, 2016

Characteristics	Frequencies	Percentage
Intention to bottle feeding		
Yes	93	27.6
No	243	72.3
Reason of intention		
Additional food will be required	56	60.2
Easy to feed	61	65.6
To back to job	63	67.7
Care givers can feed the child	76	81.7

### Factors associated with bottle feeding practice

Both bivariate and multivariate analyses were done to identify factors associated with bottle feeding practice (Table 5). The bivariate logistic regression analysis showed that there were significant associations between bottle feeding practice and age of infants/children, age of mothers, educational status of mothers, occupational status of the mothers, ANC follow up, PNC follow up and health education/counseling on bottle feeding (Table 5).

**Table 5:** Bivariate and multivariate logistic regression analysis

Variables	Bottle feeding		Crude OR (95% CI)	Adjusted OR (95% CI)
	Yes (%)	No (%)		
Age of infant/child				
0–5	9 (8.1)	102 (91.9)	0.270 (0.127,0.573)**	0.275 (0.123,0.617)*
6–11	23 (22.1)	81 (77.9)	0.869 (0.495,1.525)	0.864 (0.465,1.605)
12–23	50 (24.6)	153 (75.4)	1	1
Age of mother				
15–24	86 (74.8)	29 (25.2)	2.096 (1.036,4.237)*	3.384 (1.482,7.727)*
25–34	163 (80.7)	39 (19.3)	1.487 (0.766,2.888)	1.530 (0.740,3.163)
> = 35	87 (86.1)	14 (13.9)	1	1
Educational status of mother				
No formal education	21 (17.4)	100 (82.6)	0.560 (0.282,1.114)	0.725 (0.241,2.177)
Primary	32 (16.5)	162 (83.5)	0.527 (0.281,0.988)*	0.661 (0.237,1.841)
Secondary	21 (27.3)	56 (72.7)	1.185 (0.448,3.133)	1.419 (0.482,4.178)
Diploma and above	8 (30.8)	18 (69.2)	1	1
Occupational status of mother				
House wives	207 (88.8)	26 (11.2)	0.289 (0.173,0.484)**	0.375 (0.208,0.676)**
Other jobs	129 (69.7)	56 (30.3)	1	1
No of under 5 children				
One	14 (15.4)	77 (84.6)	1.833 (0.821,4.101)	2.791 (1.072,7.299)*
Two	52 (19.8)	211 (80.2)	1.372 (0.712,2.571)	1.449 (0.695,2.976)
Three	18 (25)	48 (75)	1	1
ANC				
Yes	71 (18.2)	319 (81.8)	1	1
No	11 (39.3)	17 (60.7)	2.907 (1.305,6.476)*	1.105 (0.409,2.981)
PNC				
Yes	55 (16)	288 (84)	1	1
No	27 (36)	48 (64)	2.945 (1.695,5.119)*	2.12 (1.083,4.139)*
Advised/counseled on bottle feeding				
Yes	30 (13)	200 (87)	1	1
No	52 (27.7)	136 (72.3)	2.549 (1.547,4.200)**	2.182 (1.241,3.835)*

*N.B*- ** *p*- value significant at level of *P* < 0.001, *p-value significant at level of *P* < 0.05

The multivariate analysis showed significant associations between bottle feeding and age of infants/children, age of mothers, number of under five children in the household, PNC follow up and advice/counseling on risks of bottle feeding.

Infants of age 0–5 months old were 72.5% times less likely to be bottle fed than children with 12–23 months old [AOR = 0.275; 95% CI: (0.123,0.617)]. Furthermore, mothers who were in the age interval of 15–24 years were 3.4 times more likely to practice bottle feeding than those who were greater than 35 years old [AOR = 3.38; 95% CI: (1.482, 7.727)]. With regard to occupational status, mothers who were housewives were 62.5% times less likely to practice bottle feeding than mothers with outside home job [AOR = 0.375; 95% CI:(0.208, 0.676)]. Mothers who have three under five children in the household were 2.7 more likely to bottle feed than those who have one under five children in the household [AOR = 2.77;95%CI:(1.07,7.14)].

In addition to this, mothers who did not attend PNC follow up were 2.1 times more likely to practice bottle feeding than mothers who had attended PNC follow up [AOR = 2.13;95%CI:(1.19,4.97)]. Furthermore, mothers who did not receive health education/counseling on risks of bottle feeding were 2.2 times more likely to practice bottle feeding than those who had received health education/ counseling [AOR = 2.18; 95% CI: (1.241,3.83)].

## Discussion

This study aimed to determine the prevalence of bottle feeding and associated factors as well as intention to practice bottle feeding in the future. About 19.6 and 27.6% mothers were bottle-feeding and had intention to practice bottle feeding during the study period respectively. Socio demographic variables like age of infant/child, age of mother, occupational status of mother and number of under five children in the household were found to be associated with bottle feeding. In addition to this, maternal health service utilization like PNC follow up and health education/counseling on bottle feeding had significant association with bottle feeding practice.

The prevalence of bottle feeding in the study area was comparable with a study conducted in other areas of Ethiopia such as Mekele town (20%) [11] and Shashemene town (20.9%) [12] and areas outside Ethiopia such as Gujarat in India (18.4%) [16]. However, the prevalence of bottle feeding recorded in this study was higher compared to the national prevalence reported in the 2011 EDHS (13%) [6], countries such as Western Uganda (10%) [17] and that reported for 43 developing countries (11%) [18]. This difference might be due to variations in socio cultural aspects among study participants.

On the other hand, the prevalence of bottle feeding recorded in this study was lower compared to those reported in some areas of Ethiopia such as southern Ethiopia (30.5%) [13], Agaro town (35%) [14], Lalibela town (28%) [15]) and other African countries such as Rural Kwazulu(23%) [19] and Kenya (22%) [20].The observed difference might be due to improved health care counseling services on IYCF including bottle feeding in our study area.

The majority 71(86.6%) of mothers who practiced bottle-feeding in the study area used cow milk and 24 (29.3%) were using cow milk for infants under 1 year. This figure is higher compared with cow milk usage reported in Agaro town 62 (78.4%) [14] and rural and semi rural women in South Africa 8(4.2%) [21]. Usage of cow milk in the study area might be associated with families’ access to cow milk as the area is known by its livestock and milk production.

The major reason for practicing bottle-feeding reported by majority of bottle-feeding mothers in current study was its easiness for feeding the child 71(86.6%). The addressed reason was different from study done in Agaro town [14], southern Ethiopia [13] and South Africa [22], in which majority of them mentioned insufficient breast milk, child did not cry when they gave bottle and it promote growth for children respectively.

In addition to this, mothers who were housewives were less likely to bottle feed than mothers who have work outside the home. The result was in line with studies conducted in Mekele town [11], Kenya [20] and Pakistan [23]. This might be rationalized as housewives have more time to breastfeed their children or to use alternative feeding methods than mothers who work outside their home.

In this study, 93(27.6%) study subjects had intention to use bottle feeding in the future. The reported result was higher than study done in England of which 7% indicating intentions to bottle-feed. High prevalence of intention in the study area might be due to lower awareness level about bottle feeding [24]. The major reason mentioned by the participants was its easiness to feed the child 61(65.6%). This similar reason was also mentioned in other studies done elsewhere in the world [24] and [25]. High prevalence of intention in the study area might be due to lower awareness level about the risks associated with bottle feeding.

As limitation this study identified both current prevalence of bottle feeding and intentions to practice bottle feeding in the future for those who were not practicing bottle feeding during the study period. However, current intention may be changed through time and can prone to inaccurate prediction despite its importance for current intervention. In addition, the study did not examine the reason why prevalence of bottle feeding varied among different socio-demographics which needs further research on the subject. However our studies identify key risk factors associated with bottle feeding.

## Conclusion

In conclusion, the prevalence of bottle feeding was higher in the study area when compared with the national prevalence whereas most of them have intention to practice it. Working outside home, lower maternal age, older age of children and more under five children in the households were found to be risk factors associated with bottle feeding practice in the study area. In addition, this study underlined the importance of postnatal care follow up and counseling on IYCF including the risks associated with bottle feeding as possible interventions for mothers/caregivers to practice appropriate IYCF in the study area.

## Abbreviations

ANC: Antenatal care
AOR: Adjusted odds ratio
CI: Confidence interval
COR: Crude odds ratio
EDHS: Ethiopian Demographic and Health Survey
ETB: Ethiopian Birr
IYCF: Infant and Young Child Feeding
PNC: Postnatal care
SD: Standard deviation
